# When targeted gene testing falls short: Case report of a novel *SLC4A3* F347S genetic variant associated with short QT syndrome

**DOI:** 10.1016/j.hrcr.2025.10.006

**Published:** 2025-10-13

**Authors:** Chelsea A. Boyd, Tyler J. Novy, Taylor A. Beecroft-Dawson, Connor A. Bell, Emily M. Bland, Christina Y. Miyake

**Affiliations:** 1 Division of Pediatric Cardiology, Department of Pediatrics, Baylor College of Medicine, Houston, Texas; 2 Department of Pediatrics, Texas Children’s Hospital, Houston, Texas; 3 Department of Molecular Physiology and Biophysics, Baylor College of Medicine, Houston, Texas; 4 Department of Molecular and Human Genetics, Baylor College of Medicine, Houston, Texas

**Keywords:** Arrhythmia, Genetics, Short QT syndrome, Cardiac arrest, Sudden death, *SLC4A3*

**Key Teaching Points**

• A normal QTc measurement in the immediate aftermath of a prolonged cardiac arrest may obscure a baseline short QTc given that the QTc interval is often relatively prolonged after cardiac arrest. In this case, consideration of Short QT Syndrome (SQTS) and subsequent follow-up QTc measurements are warranted.
• *SLC4A3* is a relatively recent genetic association with SQTS and is not included on most commercially available targeted gene panels. Additional genetic testing is often required to make this diagnosis, meriting a high index of suspicion when targeted gene panels that do not include this gene return with a negative result.
• Automated ECG platforms do not identify abnormally short QTc intervals, and this finding may go unrecognized by clinicians who are more attuned to prolonged QTc than short QTc values. It is essential that physicians consistently review the numeric QTc values and consider short QTc when values fall below the normal range, as failure to do so may result in missed or delayed diagnoses.

## Introduction

First reported in 2000, short QT syndrome (SQTS) is a rare cardiac channelopathy associated with atrial and ventricular arrythmias, high risk of sudden cardiac death (SCD), and limited treatment options. In 2013, the Heart Rhythm Society, European Heart Rhythm Association, and the Asia Pacific Heart Rhythm Society released guidelines recommending that SQTS should be diagnosed in patients with a QTc interval ≤330 milliseconds (ms) and may be diagnosed in patients with QTc interval ≤360 ms who additionally have a pathogenic variant, family history of sudden death ≤40 years old, or a prior ventricular tachycardia/ventricular fibrillation (VT/VF) episode in the absence of heart disease.^1^ Variants in 9 genes encoding ion channels have previously been associated with SQTS including *KCNH2*, *KCNQ1*, *KCNJ2*, *CACNA1C*, *CACNB2*, *CACNA2D1*, *SCN5A*, *SLC4A3*, and *SLC22A5*. However, at present, only *KCNH2* has a definitive association, and *KCNQ1*, *KCNJ2*, and *SLC4A3* have a moderate-to-strong association with SQTS.^2^ Targeted testing panels vary and typically include some or all of the following genes: *KCNH2*, *KCHJ2*, *KCNQ1, CACNA1C*, *CACNA2D1*, and *CACNB2*. Notably, most commercially available targeted genetic panels (eg, short QT panel, arrhythmia panel, comprehensive arrhythmia, and cardiomyopathy panel) do not include *SLC4A3*.

We describe a case of an adolescent male who suffered a cardiac arrest because of *SLC4A3*-related SQTS. This case is particularly notable because his QTc was never shorter than 340 ms and his pathogenic variant is not included on most commercially available short QT genetic testing panels. This highlights the need to consider SQTS even in the setting of mildly short QTc and further, to consider additional testing for *SLC4A3* when targeted genetic testing results are negative, or to seek testing panels that include *SLC4A3* when SQTS is suspected.

## Case Report

**Figure 1:**
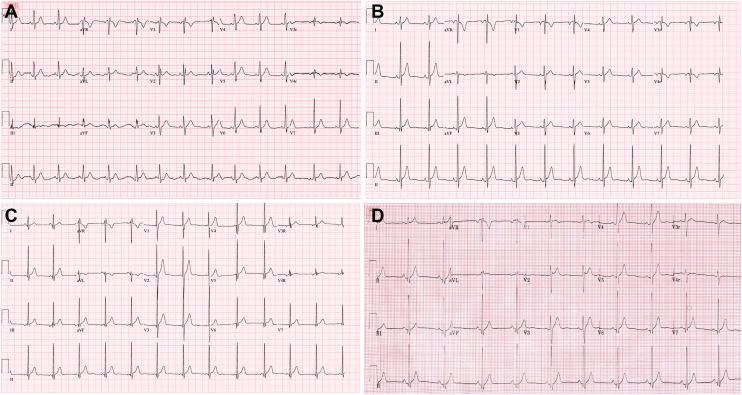
(A) Proband’s immediate post-arrest ECG, QTc 404ms; (B) Proband’s baseline ECG, QTc 348 ms; (C) Brother’s ECG, QTc 351 ms; (D) Sister’s ECG, QTc 330 ms. ECG = electrocardiogram; QTc = corrected QT.

A 17-year-old male with medical history of pectus excavatum was in his usual state of health when he collapsed while standing at the kitchen sink. The event was witnessed by family, although cardiopulmonary resuscitation (CPR) was not initiated until emergency medical services arrived. He received epinephrine, amiodarone, and a 200-joule shock for VF, although he remained in VF until another dose of epinephrine was given and a 300-joule shock was delivered. He was intubated and transported to an outside hospital where he was administered anti-epileptic medications for seizures prior to transfer to our quaternary care center. On arrival, his cardiac work-up included an electrocardiogram (ECG) demonstrating sinus rhythm and normal intervals, including a normal QTc of 404 ms (Figure 1A). There was no evidence of type I Brugada pattern. An echocardiogram demonstrated a structurally normal heart without coronary anomalies and moderately depressed biventricular systolic function that normalized by post-arrest day 2. A cardiac magnetic resonance imaging was normal and demonstrated no evidence of delayed enhancement. His ECG on post-arrest day 2 and 3 demonstrated a QTc of 364 ms and 386 ms, respectively. He had no additional arrythmias during his hospitalization and ultimately recovered without significant neurologic sequelae.

**Figure 2:**
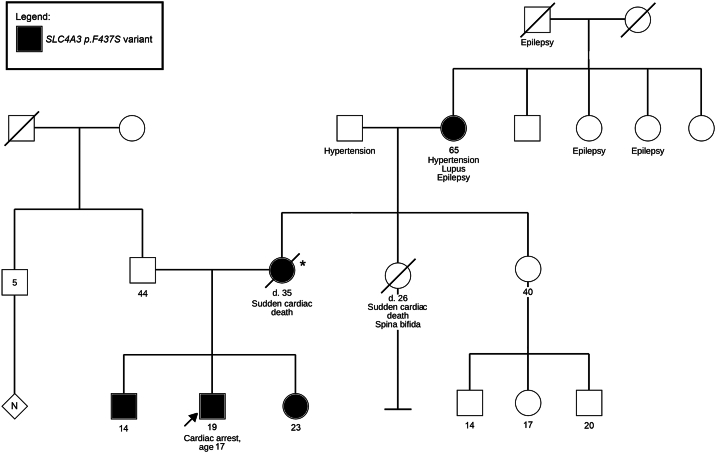
3-generation family pedigree for the index patient’s *SLC4A3* genetic variant. Notation with an *asterisk* (∗) denotes that molecular testing was unable to be obtained for the decedent, though given positive testing in her mother and her children she is assumed to harbor the same variant.

His family history was significant for sudden death—his mother, at age 35 years, who had no medical history and was in her usual state of health when she suffered a witnessed sudden cardiac arrest while seated on a couch. Autopsy was negative; genetic testing was not performed. A maternal aunt also suffered sudden death at age 26 years. She had a history of spina bifida and was recovering well in a rehabilitation facility after intestinal surgery when she collapsed while sitting in bed. Her death was attributed to post-surgical complications although there were no preceding concerns prior to her arrest, and no autopsy was performed. The only family member with prior cardiac complaints was the patient’s older sister who had long-standing complaints of chest pain and palpitations without syncope. She was evaluated by a cardiologist with an ECG that was reportedly normal. Further evaluation with a Holter or event monitor had not been performed. She declined further work-up or evaluation when offered. There was no family history of arrhythmias, including atrial fibrillation or VT. A 3-generation pedigree is shown in Figure 2; there was no paternal family history of cardiac disease or concerns. Although cardiac work-up was unrevealing, given his concerning family history a comprehensive genetic panel for inherited cardiomyopathies and arrhythmias containing 92 associated genes inclusive of SQTS (Ambry Genetics CardioNext® panel) was sent on post-arrest day 4 and resulted during his inpatient course with no identifiable variants. He had no further arrhythmias and underwent implantation of a transvenous single chamber implantable cardioverter defibrillator (ICD). Genome sequencing (GS) of the proband and the father were sent, with results pending at the time of discharge.

During his first outpatient cardiology evaluation 6 weeks post-arrest, his QTc was noted to measure short at 354 ms. At this time, A review of prior inpatient ECGs also noted a QTc of 348 ms on hospital day 19 (Figure 1B). Although GS had not yet resulted, SQTS because of SLC4A3 was suspected, and the family was urged to undergo clinical screening. All were evaluated initially at outside institutions and informed they had normal ECGs. Clinical evaluation at our center revealed a QTc of 330 ms in his older sister (Figure 1C), 351 ms in his younger brother (Figure 1D), and 404 ms in his maternal grandmother. All were suspected to have SQTS. Ultimately, the index patient’s GS resulted with a heterozygous novel variant of uncertain significance in *SLC4A3* (NM_005070.4, c.1040T>C, p.F347S, GRCh38) not harbored by the father. Family cascade testing subsequently confirmed the same variant in the maternal grandmother, patient’s sister, and his younger brother. The proband’s mother was therefore an obligate carrier for the variant, though no genetic testing had been obtained on the decedent. The variant was subsequently re-classified as likely pathogenic by the testing company (Baylor Genetics Laboratory). ICDs were discussed and offered to all family members, but at this time, only the younger brother has undergone primary prevention ICD placement. A home automated external defibrillator was recommended for all genotype-positive family members who declined primary prevention ICD. At present, 2 years post-arrest, only the index patient has had rare 3–4 beats episodes of non-sustained ventricular tachycardia on his ICD. His brother has not had any documented arrhythmias or ICD discharges. There have been no other occurrences of cardiac arrest in this family to date.

## Discussion

One of the rarest yet most malignant heritable arrhythmia syndromes, SQTS is highly associated with SCD and therefore raising clinician awareness is critical.^3^^,^^4^ This case highlights several important points in diagnosing SQTS because of *SLC4A3*.

Most pathogenic variants associated with SQTS are gain-of-function variants in genes encoding potassium channels.^5^^,^^6^ In contrast, *SLC4A3* encodes a Cl^-^/HCO_3_^-^ exchanger, with loss-of-function variants leading to increased intracellular pH and decreased intracellular chloride concentration, thereby also shortening cardiac repolarization.^7^^,^^8^ Because the commonly-tested genes associated with SQTS identify pathogenic variants in less than a quarter of cases, it is paramount to promote clinician awareness of less commonly seen clinical and genetic presentations such as presented in this case.^9^^,^^10^

Given that most cases of prolonged arrest result in prolonged QTc in the immediate post-arrest period, one should consider that a markedly normal QTc measurement in the post-arrest period may not imply a normal baseline QTc and rather might be interpreted as relatively short in this setting.^11^ Despite a prolonged resuscitation, the proband’s immediate post-arrest QTc was 404ms. In this instance, there was normalization of his baseline short QTc leading caregivers to interpret his QTc as normal and obscuring the critical underlying diagnosis. We propose that a QTc interval on the lower end of normal in the immediate post-arrest period should prompt consideration of SQTS and multiple subsequent ECGs should be performed to evaluate the QTc as the patient recovers. As noted in this patient, the QTc was relatively normal for the first 3 days post-arrest, although in retrospect, the QTc of 384 ms could have raised suspicion.

Another noteworthy feature of this case is that the patient’s baseline QTc was longer than typically seen in SQTS, with his QTc measurements after the first post-arrest day ranging from 348 ms to 386 ms. In contrast, the median QTc measurements in 3 large SQTS cohort studies were 316 ms (range 194–350 ms) and 312 ms (range: 194-355 ms); the mean QTc measurements in 2 additional large cohort studies were 314 ± 23 ms and 329 ms ± 22 ms.^4^^,^^12^, ^13^, ^14^ This is aligned with a prior cohort of SQTS patients with *SLC4A3* genetic variants in which the mean QTc was longer than might be expected in SQTS at 340 ± 18 ms.^8^ However, another recent study found QTc measurements in *SLC4A3-*related SQTS to be shorter than other SQTS genotypes with a mean QTc of 319 ± 20 ms compared with 333 ± 19 ms in the total cohort of SQTS patients.^7^ Despite contrasting literature, this case supports the notion that SQTS should still be considered when baseline QTc is borderline-normal, and adds evidence to suggest there may be reason to suspect *SLC4A3* variants in particular in these cases when the QTc is relatively long for SQTS.

Finally, although there was a family history of SCD in the mother and maternal aunt and both siblings had previously been evaluated by a cardiologist, no one in the family was accurately diagnosed until after our index patient’s cardiac arrest. Data is not available from both the siblings’ initial cardiac evaluations; however, at our institution, both were found to have short QTc intervals on ECG and genetic testing was positive for the same likely pathogenic *SLC4A3* variant found in our index patient. Although both siblings met diagnostic criteria for SQTS, it is important to note that neither the index patient nor the siblings’ short QTc measurements were flagged by the computer-generated ECG interpretation algorithm. Unlike prolonged QT intervals, short QT is not automatically flagged by automated ECG interpretation platforms, and hence the correct diagnosis relies on physicians to take active notice of the numeric measurement. Physicians are routinely trained to recognize and report long QTc intervals, but the same practice rarely applies to short QTc intervals. Diagnosis in this patient and his family additionally required a high index of suspicion for SQTS and the awareness that additional genetic testing was necessary because of the incomplete nature of most commercially available SQTS gene panels at present. Physicians must take notice of abnormally short QTc values, be aware that there are limitations to targeted gene panels, and have the knowledge that additional genetic associations, such as *SLC4A3,* require additional testing if they are not included in targeted panels. We believe that *SLC4A3* should be incorporated into all targeted gene panels for SQTS.

## Conclusion

Despite the rarity of SQTS, it is critical to increase clinician awareness of the spectrum of clinical findings and genetic associations in this condition, as this may lead to life-altering diagnoses for patients and their families. Cases such as this not only heighten clinician awareness of SQTS but highlight a few additional key points. First, although the typical range of QTc measurements in SQTS is generally thought to be less ≤330 ms, patients with this condition may have normalization of the QTc in the post-arrest period, may have borderline-normal QTc measurements at baseline, and ultimately some may never have a QTc measurement below 340 ms. Maintaining a high index of suspicion is important in these cases to make the diagnosis. Second, when clinical suspicion is present, targeted gene testing may prove insufficient and further genetic testing must be considered until genetic panels are inclusive of *SLC4A3*. Finally, accurate diagnosis is critical not only for the management of the index patient, but for identification and treatment of family members at risk. It is critical to continue to reevaluate patients and families with clinical suspicion for heritable arrhythmia syndromes, even when prior cardiac evaluations have been reported to be unrevealing, recognizing that our knowledge about these rare conditions is continually growing, as demonstrated in this case.

## Disclosures

Dr. Christina Miyake – Advisor, Advancing Women in EP, Boston Scientific. All other authors have no conflict of interests to disclose.
